# Seeing red..? Co-design of a culturally tailored uterine cancer awareness intervention for Asian and Black ethnic minority groups

**DOI:** 10.1016/j.gore.2025.101690

**Published:** 2025-02-07

**Authors:** A. Chitrakar, N. Darko, E.L. Moss

**Affiliations:** a College of Life Sciences, University of Leicester, Leicester, LE2 7LX, UK; b University Hospitals of Leicester NHS Trust, Infirmary Square, Leicester, LE1 5WW, UK

**Keywords:** Uterine cancer, Endometrial cancer, Womb cancer, Red-flag, Symptoms

## Abstract

**Objective:**

There is a lack of public awareness of the signs and symptoms of uterine cancer, and common misconceptions can lead to delays in diagnosis.

**Methods:**

A three-step co-design principle was undertaken to iteratively develop a culturally- tailored uterine cancer awareness animated video for Asian and Black ethnic minority populations. A series of focus groups were held with representatives from the target populations, uterine cancer survivors and healthcare professionals to firstly review the storyboard and script, and secondly to review the animated video and make adaptions. The content of focus group discussions and iterative changes were analysed using thematic reflexive analysis.

**Results:**

In total, 39 individuals participated in the co-design process, including 21 women from Asian and Black minority groups, four uterine cancer survivors and seven healthcare professionals, including three gynaecologists, two specialist nurses and two general practitioners. The key themes identified included lack of awareness, clarity of message, visual appearance and cultural tailoring, factual accuracy, and addressing misconceptions. The results emphasised the importance of co-designing health communication materials collaboratively with individuals from underserved ethnic minority groups, healthcare professionals, and uterine cancer survivors.

**Conclusions:**

The created animated video conveys key health messages by addressing critical themes including awareness gaps in reproductive anatomy, uterine cancer symptoms, and misconceptions about diagnostic procedures, whereas the use of diverse visual representations and simple language aims to foster inclusivity and cultural resonance.

## Highlights

• Co-design of a culturally tailored information video with members of the public, health professionals, and cancer survivors.
• ‘Seeing red..?’ addresses patients’ information requirements in an equitable and accessible format.
• ‘Seeing red..?’ can be used to raise awareness of uterine cancer amongst underserved and ethnically diverse populations.

## Graphical abstract

**Figure:**
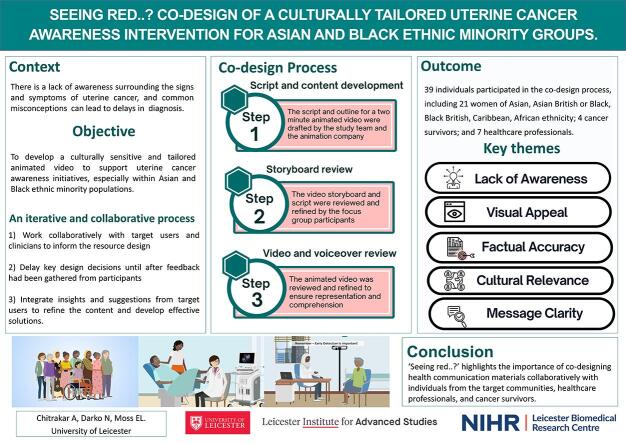

## 1 Introduction

Uterine (womb/endometrial) cancer is now the most common gynaecological cancer in many countries, including in the UK (England/Scotland/Wales/Northern Ireland) with nearly 10,000 new cases/year. Uterine cancer incidence in the UK is rising, increased by 58 % over the past three decades (Cancer Research, 2023), and this is coupled with an increasing likelihood of dying from uterine cancer, a median increase in mortality rate of 31.7 % between 2002 and 2019 (Rashid et al., 2024). The rising incidence of uterine cancer has been attributed to the increasing prevalence of risk factors, in particular obesity, physical inactivity and a fall in hysterectomy rates for benign disease (Mohammed et al., 2021, Cancer Research, 2023). The impact of uterine cancer on women from ethnic minority populations is disproportionately greater than for White ethnicity women, with women from Black ethnic groups in the UK having a 2.5 times greater mortality rate (Moss et al., 2023). Additionally, data from the USA has identified that Asian ethnicity women have a 3.4 times higher incidence of uterine cancer compared to White populations, with the largest incidence increases being seen in women from Indian and Pakistani populations (Johnson et al., 2024). Women from Black Caribbean and African groups resident in the UK are more likely to present with advanced-stage uterine cancer (Fry et al., 2023), thereby contributing to significantly worse survival rates. Researchers in the USA have been reporting disparities in uterine cancer outcomes amongst women from Black ethnicity populations for over a decade (Clarke et al., 2022). Knowledge gaps and misattribution of red-flag symptoms, in particular vaginal bleeding, have been reported by endometrial cancer survivors of Black ethnicity (Doll et al., 2020). These findings were also shown in our previous research with individuals from ethnic minority groups living in the UK, which identified that many women do not seek medical review when experiencing red-flag uterine cancer symptoms, for example postmenopausal vaginal bleeding, due to a lack of awareness, normalisation of symptoms, or embarrassment (Kumarakulasingam et al., 2018). Instead, many individuals utilise the lay healthcare system within their ethnic community, asking advice from family or friends (Liu et al., 2015), which can lead to a delay in diagnosis and potentially impact uterine cancer survival.

A common critique of health promotion efforts is that they aim to serve dominant groups through the application of traditional behavioural approaches, which have neglected the multiple social and political drivers of health (Baum and Fisher, 2014). Typically, communication of health-related information tends to rely on the persuasiveness of statistical information or on strategies which highlight the benefits or negatives of a particular behaviour to promote or advise against that behaviour (Zebregs et al., 2015). Instead, information in the form of stories or pictorial can be culturally ubiquitous as it has the ability to cut through intersections of socio-economic position and culture (Houts et al., 2006, Kreuter et al., 2007). Working with target communities is a prerequisite for developing information materials and specifically the principle of co-design, which involves the “active collaboration between stakeholders in designing solutions to a prespecified problem” (Vargas et al., 2022) can be used to ensure the information is culturally appropriate and relatable.

Targeted awareness, as exemplified by Black men and prostate cancer awareness (NHS England,2018), has shown that collaborative community engagement can have a sustained impact on cancer mortality, with Black men in the UK now being less likely to be diagnosed at a late stage as compared to men from other ethnic groups (Fry et al., 2023). However, the lack of culturally sensitive, tailored information resources for other cancer types can create additional barriers, particularly with regard to language and representation. Low levels of health literacy can result in difficulty understanding medical terms or the benefits of early diagnosis, leading to further delays in accessing care, and has been identified in many studies as being associated with poor cancer outcomes, as individuals are less likely to be involved in screening or medical care (Berkman et al., 2011, Davis et al., 2002). Ethnic minority women who participated in our previous studies (Kumarakulasingam et al., 2018, Darko et al., 2023) expressed a need for culturally sensitive and tailored resources, emphasizing the importance of addressing common red-flag symptoms and dispelling culturally accepted myths within their communities. They identified a need for clear, straightforward messaging and preferred animated videos, over traditional spoken formats, since they were viewed as being more eye-catching and could be distributed through social media and instant messaging channels, ensuring accessibility and reach. The aim of the project was to co-design an animated uterine cancer awareness video with women of Black and Asian ethnicity, uterine cancer survivors, and healthcare professionals to directly address the informational needs identified during our research.

## 2 Methods

Ethical approval was granted for the study by the University of Leicester Medicine and Biological Sciences Research Ethics Committee (39250).

**Fig. 1:**
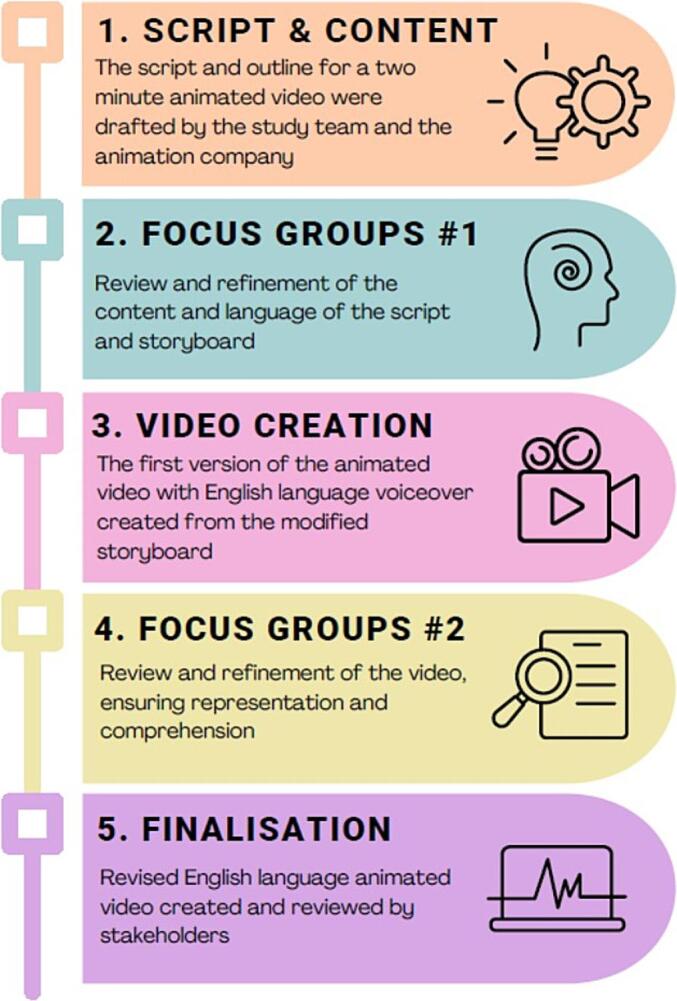
The co-design process for ‘Seeing red..?’.

An information intervention (an animated video), underpinned by Social Cognitive Theory (SCT) (Bandura and National Institute of Mental Health 1986) and the Health Belief Model (HBM) (Rosenstock et al., 1988), was designed using the co-design principle involving an iterative and collaborative process. End-users and community partners were engaged at multiple stages to tailor and refine the video through discussion and feedback. This approach involved: a) collaborating with both target users and clinicians to inform the resource design; b) delaying key design decisions until after feedback had been gathered from participants; and c) integrating the insights and suggestions from target users to refine the content and develop effective solutions. A three-step co-design principal process (Vargas et al., 2022) was followed by working with the animation company Science Animated (https://sciani.com) (Fig. 1). Firstly, an outline script and storyboard to define the content and key messages for a 2-minute animated video were developed through an iterative process between primary and secondary care clinicians involved in the care of uterine cancer, and a uterine cancer survivor. The video content was determined *a priori* from previous consultation work and was adapted to cover the reported information needs, including red-flag symptoms, what to do if symptomatic, risk factors, and addressing commonly held misconceptions.

Secondly, the created storyboard was reviewed, frame-by-frame, in a series of focus groups with women from different ethnic minority groups, uterine cancer survivors and healthcare professionals. Demographic information, such as age, gender, and ethnicity, was requested from the participants. Changes advised by the focus groups were made to the storyboard, and further focus groups were held until no further changes were advised. The storyboard was then created into an animated video, and further rounds of focus groups were held to review and revise the visual appearance and messaging within the video. The focus groups continued until no further changes were advised, with the iterative nature of the process reflecting the co-design principle. Finally, individuals involved in the cancer awareness or cancer diagnostic services were invited to participate in a focus group or a one-to-one interview to review the finalized video for their comments and feedback.

Participants were recruited through the NIHR Centre for Ethnic Health Research, University of Leicester, the Leicester NIHR BRC Public Involvement Team, Peaches Womb Cancer Trust, and established collaborations with community groups within the East Midlands region of England. The focus groups were conducted virtually using Microsoft Teams, lasted one-hour in duration and were facilitated by a member of the research team (EM/AD/AC). The discussions were recorded and transcribed, and field notes were taken. Analysis was performed by a researcher (AC) with qualitative research experience using thematic reflexive analysis (Braun and Clarke, 2023). Open codes were assigned to the data with explanatory notes. To ensure inter-coder reliability, two additional researchers (ND/EM) independently reviewed 3–4 transcripts, and their codes were compared with those of the first researcher. Any discrepancies were resolved through discussion, and an agreed set of codes was established to form the initial analytic framework. Further transcripts were reviewed, and the analytic framework was applied. New codes that were identified were added, and the analytic framework was revised accordingly until no new codes emerged. Once all transcripts had been revised, the final analytic framework was applied to all the transcripts and reviewed for connections between participants and categories. Throughout the process, the researchers engaged in reflexive practice, considering how their own perspectives may have influenced the interpretation of the data to ensure objectivity and that the findings authentically reflected participants’ experiences, and to reduce unconscious research bias. Additionally, peer debriefing sessions were held with other research team members to review and refine the identified themes.

## 3 Results

In total, 39 individuals participated in the co-design process, including 21 women from Asian, Asian British or Black, Black British, Caribbean, African minority groups (15 and 6, respectively), four uterine cancer survivors, and seven healthcare professionals, including three gynaecologists, two specialist nurses and two general practitioners (Table 1). Three focus groups with 16 participants were held at stage 1 (co-design steps 1 and 2), reviewing and iteratively refining the proposed storyboard, and 18 individuals participated in 10 focus groups at stage 2 (co-design step 3), reviewing and iteratively changing the animated video. Five participants from the target audience, three healthcare professionals and all four cancer survivors contributed at both the storyboard and video reviewing stages. In addition, the created video was reviewed by seven staff members from cancer awareness charities or cancer services to discuss potential avenues for dissemination and referral pathways for patients presenting with symptoms.

**Table 1:** Demographics of individuals from the target population and uterine cancer survivors who contributed to the co-design focus groups. Information on age was provided by 13 of the 22 participants.

**Demographic characteristics**	**Number of focus group participants**
**Individuals from Asian and Black ethnic populations**	
Median age	49 years,
Age range	range 34–71 years
Ethnicity:	
**Asian/Asian British – total**	**15**
Asian, Asian British: Indian	8
Asian, Asian British: Pakistani	1
Asian, Asian British: Bangladeshi	2
Asian, Asian British: Other Asian	1
Asian, Asian British: Not specified	3
**Black, Black British, Caribbean or African − total**	6
Black, Black British: African	3
Black, Black British: Caribbean	1
Black, Black British: Not specified	1
Mixed or Multiple ethnic groups: White and Black Caribbean	1
**Uterine cancer survivors**	
White British	2
White European	1
Not disclosed	1

Analysis of the focus group discussions identified several key themes that informed the content and production of the video: 1) Lack of Cancer Awareness, 2) Clarity of Messaging, 3) Visual Appearance and Cultural Tailoring, 4) Factual Accuracy and Addressing Misconceptions, and 5) Dissemination of Health Information and Future Communication Strategies.

1) Lack of awareness

**Fig. 2:**
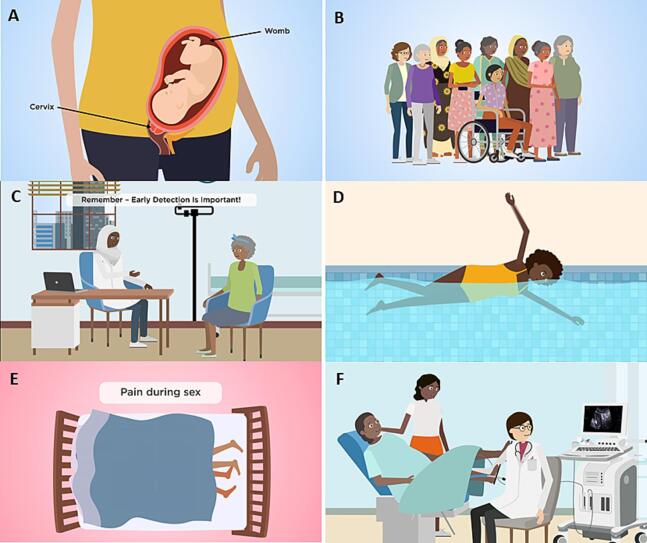
Images from the ‘Seeing red..?’ animated video. 2A) Image of the uterus carrying the pregnancy and labels highlighting the anatomical difference between the uterus and cervix; 2B) Image of women from different ethnic, age and health backgrounds; 2C) Image of a healthcare professional and patient from ethnic minority populations; 2D) Image of a young Black ethnicity woman swimming; 2E) A gender-neural image to represent dysparunia; 2F) Image of a patient undergoing a transvaginal ultrasound scan.

Many participants from an Asian ethnic background were unaware of the high incidence of uterine cancer in the UK and were surprised at the lack of health information compared with cervical and ovarian cancer. The Black and Asian women reported a lack of awareness of the signs and symptoms of uterine cancer, but a number of also reported very limited knowledge of reproductive anatomy. As a result, the need to include an introductory scene identifying the uterus (e.g., as carrying a pregnancy) and differentiating it from the cervix (Fig. 2A) was advised, thereby helping viewers to understand the difference between the uterine cancer and cervical cancer.

There was considerable discussion regarding terminology amongst all participants and the need to include plain English language descriptions when referring to the uterus. The term ‘womb’ was preferred by all the participants since it was felt that this term was better understood by the general population and by those with limited health literacy, compared to terms such as ‘uterus’ or ‘endometrium’. However, one of the health professionals noted that medical terms were also required to avoid confusion: “*People don't know anatomical terms, so simpler terms, with pictures that have labels on them, should be used*”.

Participants highlighted that technical and formal communication of health messages could make the message confusing or disconnected, especially amongst non-English-speaking communities. This was discussed further when the potential of creating multi-lingual versions of the video was discussed. Language was identified as potentially creating barriers in understanding, particularly the use of formal medical terms in another language. “*If you use medical terminology, even in Gujarati it will mean nothing. It's just mumbo jumbo. We use simple everyday words that people use in conversation with each other, not just in a written translation.”* Female Asian participant.

2) Clarity of message

Participants discussed several possible titles for the video to ensure clarity in the message. Including the word “red” was preferred since it represented danger and was perceived as an effective way of emphasising abnormal bleeding as a warning sign. The title “Seeing Red..?” was eventually chosen, with one participant noting, “*Seeing red is always kind of associated with danger.”* Female Asian participant. The idiomatic connotations of ‘seeing red’ referring to toward feelings of anger was raised in focus group discussions by several women whose primary language was English. Alternative titles were discussed within the groups, for example ‘red spot’, however given that a ‘bindi’, a Hindu symbol worn on the forehead by women, is a red dot it was felt that this was an inappropriate title.

All groups emphasised that the video needed to convey a “*very simple message”*. Participants preferred a positive, encouraging tone rather than one that instilled fear, leading to several revisions of the video script. The phrase “early diagnosis means more easily treated” was considered more motivating and accessible compared to the original proposed wording, ‘*early diagnosis equals better outcomes.’* As one healthcare professional remarked, “*yeah, early diagnosis. At least that’s better—it’s more easily treated the earlier it’s found, isn’t it? Something along those lines to encourage people at the early stage.”* Another participant, from a Black ethnic minority group also felt the message was clear and effective for raising awareness, noting, “*Early diagnosis, quick treatment. The language is simple and easy to understand.”*.

Despite the volume of information on the video, participants expressed the belief that it did not detract from the main message. This was summed up by an Asian ethnicity participant: “*The main takeaway for me from this* video *is if I bleed when I shouldn't, then I know I need to…go and see my doctor.”*.

Participants also appreciated the speed and tone of the narrator, including those who were not primary English speakers, as this made the delivery slow and precise enough to follow through with the video. “*The pace of the lady speaking was quite good. I thought the speaker's pace was really effective,”* Asian ethnicity focus group participant. Another participant, who has dyslexia, appreciated the clarity of the presentation “*Even with my learning difficulty, the information was clear and easy to understand.”*.

3) Visual appearance and cultural tailoring

Combining clear messaging with eye-catching visuals that featured diverse characters was seen as essential for engaging people from different ethnic backgrounds and underserved groups. This included portraying a range of ethnicities and dress styles for both patients and healthcare professionals (Figs. 2B and 2C). As one Asian ethnicity participant observed, “*I actually liked the characters used. There was a Muslim woman as the doctor, and then an older woman. It cuts across all ethnicities*.” The participants also valued seeing diverse characters in the video, both in terms of ethnicity and age. As one Black ethnic minority participant said, “*I like that there’s people that look like me, but also a diverse range. Not just in race, but also age.”.*

Changes to scenes were made in response to comments from the focus groups with respect to cultural and religious sensitivities. For example, the position of a character was changed to maintain modesty, standing in a bathroom rather than sitting on a toilet with her legs uncovered. Such details were revised several times to ensure that the visuals were both inclusive and respectful.

This was also valued by the participants, who noted how such interactions helped balance some racial stereotypes and break down preconceived ideas of other groups to favour inclusion. One such example was a swimming scene with a Black woman, which a Black ethnic minority participant appreciated: “*Of course, it is OK. To see Black people swimming*.” Another Black ethnic minority participant noted that swimming was not common in older generations within her community: “*For us, swimming isn't common, especially for older generations, so it doesn't feel real*.” With this in mind, the woman featured in this scene was changed to a young Black woman to reflect the growing engagement of younger generations in activities such as swimming and to provide a more realistic image (Fig. 2D).

One scene that was challenging to visualise for both Black and Asian minority women was ‘pain during intercourse’ due to the sensitive nature of the symptom. Several revisions were undertaken, and the agreed version was for only a couple’s feet to be seen (Fig. 2E), as this was a compromise between modesty and gender neutrality. “*Another way could be just having feet-you just see feet*.” Asian ethnicity participant.

4) Factual accuracy and addressing misconceptions

It is essential that health communication is not only culturally appropriate but also factually accurate. This topic emerged in the focus groups, particularly regarding the initial investigation typically performed for suspected uterine cancer: a transvaginal ultrasound scan. Healthcare professionals stressed the importance of including this in the video, as patients may be unaware of the procedure and might refuse if they attended an appointment unprepared. As one participant explained, “*how to prepare for..*[and] *what to expect when you go to your appointments”* was crucial. It was agreed that demystifying the procedure, particularly by showing the presence of a chaperone, could help alleviate fears around the procedure or scan (Fig. 2F). For example, one participant noted, “*The chaperone in the pictures is fantastic because sometimes people can feel a bit daunted going on their own.”* Asian ethnicity participant.

The previously identified misconceptions surrounding uterine cancer were confirmed in the discussions held with women from the target population. Several participants reported surprise upon being told that a cervical smear test did not detect uterine cancer and that even a small amount of post-menopausal bleeding should prompt medical review. For example, one Black ethnic minority participant stated: “*A couple of things surprised me, but one was that the smear test doesn't pick it up!*”.

5) Dissemination of health information and future strategies for communication

Participants expressed a strong desire to receive health education resources through multiple platforms that can effectively reach their communities. Many identified social media and messaging platforms, such as WhatsApp, as key avenues for sharing information and engaging with a larger audience. One Black ethnic minority participant noted, “*People in our [African] communities share it on WhatsApp, group chats, and spaces like that, more so with Black women groups.”*. Another participant from an Asian ethnic minority group added. *“We need this kind of content to circulate on social media more.”*.

It was emphasised that campaigns should address specific health concerns within Asian and Black minority communities. For example, one participant suggested, “*Include topics like menopause, which some communities may know very little about.”* Black ethnicity participant. The challenge of targeting women within the typical uterine cancer age group (over 55 years) without causing undue anxiety among younger women was also discussed. Healthcare Professionals also expressed concern about the potential impact on primary care services, with one stating, “*You could get a lot of people just seeing that one symptom and then going to their GP saying, ‘I think I’ve got womb cancer,’”* and another added “*I know when I tried to do something similar to this* [raise awareness*], they’re like,* “*You can’t put this because we’ll overwhelm* [the system].”.

## 4 Discussion

It is essential that impactful information campaigns that result in sustained cancer symptomatology awareness and improve health literacy amongst individuals from underserved populations are collaborative partnerships and reflect the voices and imagery of the target audiences (Alson et al., 2021). The methods of co-creation, co-design, and co-production all include collaborative working with participants from the communities of interest and emphasise the importance of tailoring interventions to cultural values, population needs and community engagement (Oluloro et al., 2024). ‘Seeing red..?’ utilised a co-design methodology since the plan for an animated video was determined *a priori* through previous work (Darko et al., 2023), and there was a step-by-step process where representatives from end-user groups participated in the design stage to develop an information video that addressed the user's preferences and expectations (Sanders and Stappers, 2008). This co-design approach with community representatives, as utilised in this study, enables their voices and opinions to shape the appearance and messaging, to create resources that can be used with maximum impact. In particular, the numerous iterative changes of the video script, characters and content resulted in cultural-tailoring through embedding representation and being sensitive to cultural issues and sensitivities.

Interventions designed to influence health beliefs and behaviour should have a theoretical foundation and be embedded within a socio-cultural framework. This project was based upon two theories: firstly, SCT, the influence of intersectional factors, including personal characteristics and environment impact behaviour; and secondly, the HBM, which includes the influence of perceived severity, susceptibility and barriers on health behaviours. The cultural adaptation of ‘Seeing red..?’ was designed to better fit with the expectations of women from Asian and Black ethnic minority groups, with the aim of decreasing disagreement and increasing acceptance (Bandura and Health NIoM, 1986, Rosenstock et al., 1988). Numerous studies of various healthcare interventions undertaken with different populations have evidenced the importance of partnering with end users to maximise impact (Voorber et al., 2015), since such methods can identify the optimum delivery methods that resonate with the target audience's beliefs and values (Kreuter et al., 2003). This can lead to the target audience being more likely to follow the advice, and consequentially the intervention will have a more significant effect (Swanepoel, 2003).

Previous studies have shown that simple messaging, together with easy-to-understand illustrations, can address deficiencies in anatomical and reproductive knowledge (Gotlieb et al., 2022). Examples include a culturally adapted prostate cancer awareness game aimed at African Caribbean men that produced positive changes in health literacy (Cosma et al., 2015). It was viewed by participants as being particularly effective because it had ‘been designed by the Community for the Community’ and increased intentions to seek medical review in the event of symptoms. The efficacy of cultural competency within information resources was demonstrated with the wMammogram intervention, an app-based mammogram intervention aimed at women from the Northern Plains American Indian population (Roh et al., 2023), where receiving culturally appropriate information resulted in a significantly higher uptake of breast screening and a greater willingness to disseminate information to peers compared to a standard information brochure. Roh’s study also highlighted a preference for the dissemination of information through digital pathways rather than printed information; a view echoed in our and other studies (Lenoir et al., 2017).

Cultural representation is an important factor in health communication strategies, and the use of minority characters in health stories helps to remove prejudice since it presents minority groups in a more relatable light, thereby aiming to generate a better response from such groups (Nair and Adetayo, 2019). Many of the focus group participants identified characters within the video that reflected their ethnicity and/or culture, and this was felt to increase their positivity towards the video and echoes the findings from other studies in different populations (Resnicow et al., 1999). Tailoring content to ensure cultural and language competency is reported to result in greater acceptance by consumers, particularly from minority groups (Betancourt et al., 2003). In ‘Seeing red..?’, the breadth of representation was not confined to ethnic diversity but included a range of age and body habitus within the characters, which is particularly important since uterine cancer has significant associations with increasing age and obesity. This was viewed as an additional method for attracting the attention of women with these risk factors. The impact of seeing one’s own characteristics reflected in a cancer narrative is exemplified by the ‘Jade Goody Effect’ following the death of the TV personality Jade Goody from cervical cancer in 2009 at the age of 27 years. This resulted in a 70 % higher attendance than expected and 478,000 additional screening attendances, particularly in younger women (under 50 years) who did not engage regularly with screening (Lancucki et al., 2012) and were from lower socioeconomic groups (Marlow et al., 2012).

Further evaluation will be needed to determine the impact of the ‘Seeing red…?’ video on women’s health-seeking behaviour intentions and the impact on clinical services. General cancer awareness campaigns have resulted in large increases in the demand for primary/secondary care appointments and investigations (Eberhardt et al., 2022). For example, the ‘Be Clear on Cancer’ campaigns have resulted in increase in referrals to specialist services but this may not convert into increased cancer diagnoses (Mazari et al., 2017) or have had a very short-lived impact of only a few months (Bethune et al., 2013). National cancer awareness campaigns have a greater impact on people from higher socioeconomic groups (Hall et al., 2016) and it is acknowledged that campaigns aimed at addressing barriers experienced by individuals from ethnic minority populations should be culturally sensitive and targeted (Niksic et al., 2016). It is anticipated that the co-designed ‘Seeing red..?’ resources can be used for such a targeted campaign in the future.

### 4.1 Strengths and Limitations

The key strength of this study is the co-design process enabling a broad range of cultural perspectives with different experiences, including from diverse age, ethnicity and religious populations. There is no minimum number of participants required for undertaking such collaborative work, and we cannot say that we were able to capture the full diversity of the Asian and Black ethnic populations resident within the UK, since these are not homogeneous communities and potentially have different health beliefs and practices. The number of focus groups at the video reviewing stage was increased in order to obtain a wider range of opinions to be voiced and additional cultural perspectives to be considered, reflecting the need to continue data collection until data saturation was reached. Further work to validate the study findings and the views expressed by the study participants is planned amongst larger groups of women from ethnic minority populations and to explore the requirements for an awareness campaign based on the ‘Seeing red..?’ resources.

## 5 Conclusion

This study highlights the importance of co-designing health communication materials collaboratively with individuals from the target populations, healthcare professionals, and cancer survivors. The collaborative approach allowed for the creation of culturally sensitive and tailored resources addressing the information needs of women from ethnic minority populations and those with lower health literacy. The created animated video conveys key health messages by addressing critical themes including awareness gaps in reproductive anatomy, uterine cancer symptoms, and misconceptions about diagnostic procedures, whereas the use of diverse visual representations and simple language aimed to foster inclusivity and cultural resonance. Further work is now needed to determine the impact of the ‘Seeing red..?’ on increasing uterine cancer awareness and the health-seeking behaviour of women with red-flag symptoms**.**

**Funding**

This is a summary of independent research funded by a Leicester Institute for Advanced Studies Pioneering Partnerships grant and carried out at the National Institute for Health and Care Research (NIHR) Leicester Biomedical Research Centre (BRC). The views expressed are those of the author(s) and not necessarily those of the funder, the NIHR or the Department of Health and Social Care.

## CRediT authorship contribution statement

**A. Chitrakar:** Writing – review & editing, Writing – original draft, Formal analysis. **N. Darko:** Writing – review & editing, Writing – original draft, Validation, Supervision, Methodology, Investigation, Funding acquisition, Formal analysis, Conceptualization. **E.L. Moss:** Writing – review & editing, Writing – original draft, Validation, Supervision, Project administration, Methodology, Investigation, Funding acquisition, Formal analysis, Data curation, Conceptualization.

## Declaration of competing interest

The authors declare the following financial interests/personal relationships which may be considered as potential competing interests: Dr Moss and Dr Darko have received research funding from the North East London Cancer Alliance, the Leicester Leicestershire and Rutland Integrated Care Board, British Gynaecological Cancer Society and The Eve Appeal. Dr Chitrakar has received support from North East London Cancer Alliance and the Leicester Leicestershire and Rutland Integrated Care Board for unrelated research projects.

## Data Availability

The participants of this study did not give written consent for their data to be shared publicly, so due to the sensitive nature of the research, supporting data is not available.
